# Supplementary material for: Causal Relationship between Aging and Anorexia Nervosa: A White-Matter-Microstructure-Mediated Mendelian Randomization Analysis
Source: Biomedicines. 2024 Aug 16;12(8):1874. doi: 10.3390/biomedicines12081874 (PMC11351342; doi:10.3390/biomedicines12081874)
Supplement: Supplementary file 1 [file biomedicines-12-01874-s001.zip › Supplementary Figure/Figure S4/Singe SNP plot of MR analysis for TL to ubm-b-1988.pdf]

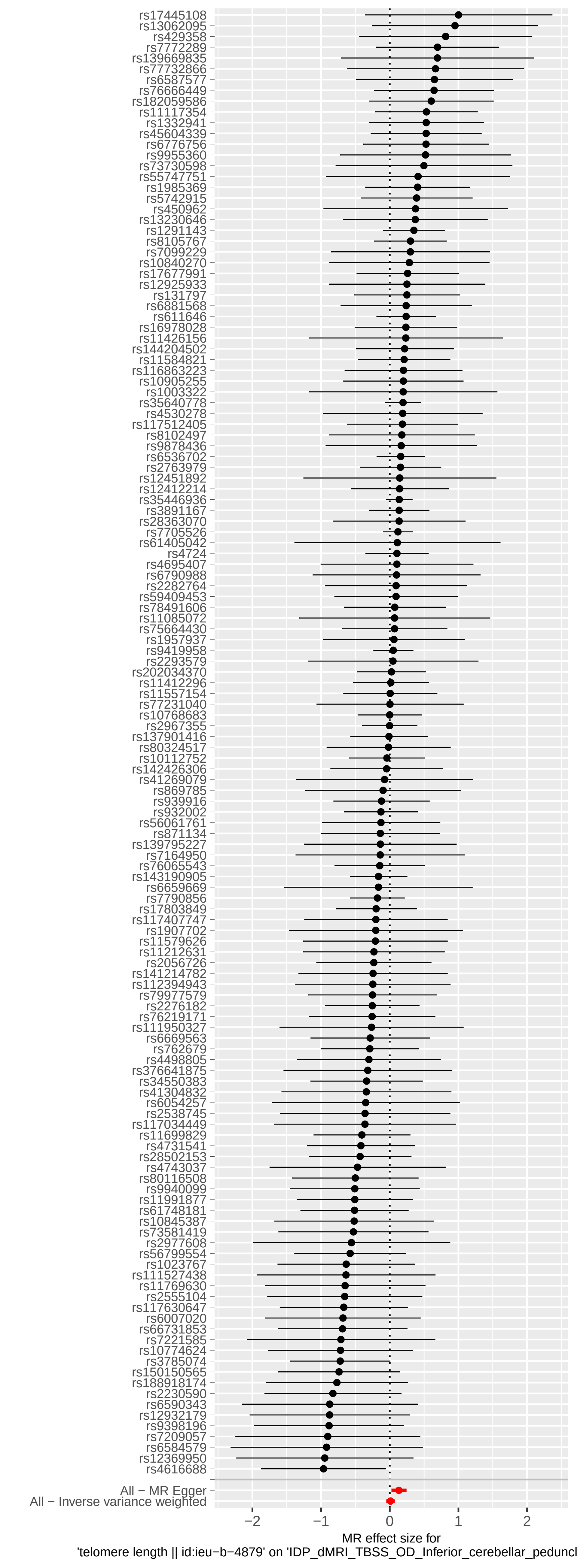
rs17445108
rs13062095
rs429358
rs7772289
rs139669835
rs77732866
rs6587577
rs76666449
rs182059586
rs11117354
rs1332941
rs45604339
rs6776756
rs9955360
rs73730598
rs55747751
rs1985369
rs5742915
rs450962
rs13230646
rs1291143
rs8105767
rs7099229
rs10840270
rs17677991
rs12925933
rs131797
rs6881568
rs611646
rs16978028
rs11426156
rs144204502
rs11584821
rs116863223
rs10905255
rs1003322
rs35640778
rs4530278
rs117512405
rs8102497
rs9878436
rs6536702
rs2763979
rs12451892
rs12412214
rs35446936
rs3891167
rs28363070
rs7705526
rs61405042
rs4724
rs4695407
rs6790988
rs2282764
rs59409453
rs78491606
rs11085072
rs75664430
rs1957937
rs9419958
rs2293579
rs202034370
rs11412296
rs11557154
rs77231040
rs10768683
rs2967355
rs137901416
rs80324517
rs10112752
rs142426306
rs41269079
rs869785
rs939916
rs932002
rs56061761
rs871134
rs139795227
rs7164950
rs76065543
rs143190905
rs6659669
rs7790856
rs17803849
rs117407747
rs1907702
rs11579626
rs11212631
rs2056726
rs141214782
rs112394943
rs79977579
rs2276182
rs76219171
rs111950327
rs6669563
rs762679
rs4498805
rs376641875
rs34550383
rs41304832
rs6054257
rs2538745
rs117034449
rs11699829
rs4731541
rs28502153
rs4743037
rs80116508
rs9940099
rs11991877
rs61748181
rs10845387
rs73581419
rs2977608
rs56799554
rs1023767
rs111527438
rs11769630
rs2555104
rs117630647
rs6007020
rs66731853
rs7221585
rs10774624
rs3785074
rs150150565
rs188918174
rs2230590
rs6590343
rs12932179
rs9398196
rs7209057
rs6584579
rs12369950
rs4616688
All − MR Egger
All − Inverse variance weighted
−2
−1
0
1
2
MR effect size for
'telomere length || id:ieu−b−4879' on 'IDP_dMRI_TBSS_OD_Inferior_cerebellar_peduncl
